# Cognitive improvement effects of PF-04957325, a phosphodiesterase-8 inhibitor, in mouse models of Alzheimer’s disease via modulating neuroinflammation

**DOI:** 10.1093/ijnp/pyaf028

**Published:** 2025-05-02

**Authors:** Tian-yang Guo, Meng Zhang, Yu-li Lv, Nian-zhuang Qiu, Rui-min Chen, Fang-fang Zhang, Wei Chen, Feng Zhang, Yong-feng Gao, Xiao-dan Wang, Xue-hui Zhang, Mei-hua Chen, Han-ting Zhang, Hao Wang

**Affiliations:** Institute of Pharmacology, Shandong First Medical University & Shandong Academy of Medical Sciences, Tai’an, Shandong, China; School of Pharmacy, Key Laboratory of Molecular Pharmacology and Drug Evaluation (Yantai University), Ministry of Education, Yantai University, Yantai, China; State Key Laboratory of Advanced Drug Delivery and Release Systems, Shandong Luye Pharmaceutical Co., Ltd., Yantai, Shandong, China; Institute of Pharmacology, Shandong First Medical University & Shandong Academy of Medical Sciences, Tai’an, Shandong, China; Institute of Pharmacology, Shandong First Medical University & Shandong Academy of Medical Sciences, Tai’an, Shandong, China; Institute of Pharmacology, Shandong First Medical University & Shandong Academy of Medical Sciences, Tai’an, Shandong, China; Institute of Pharmacology, Shandong First Medical University & Shandong Academy of Medical Sciences, Tai’an, Shandong, China; Institute of Pharmacology, Shandong First Medical University & Shandong Academy of Medical Sciences, Tai’an, Shandong, China; Institute of Pharmacology, Shandong First Medical University & Shandong Academy of Medical Sciences, Tai’an, Shandong, China; Institute of Pharmacology, Shandong First Medical University & Shandong Academy of Medical Sciences, Tai’an, Shandong, China; Institute of Pharmacology, Shandong First Medical University & Shandong Academy of Medical Sciences, Tai’an, Shandong, China; Institute of Pharmacology, Shandong First Medical University & Shandong Academy of Medical Sciences, Tai’an, Shandong, China; Institute of Pharmacology, Shandong First Medical University & Shandong Academy of Medical Sciences, Tai’an, Shandong, China; Institute of Pharmacology, Shandong First Medical University & Shandong Academy of Medical Sciences, Tai’an, Shandong, China; Department of Pharmacology, Qingdao University School of Pharmacy, Qingdao, Shandong, China; Institute of Pharmacology, Shandong First Medical University & Shandong Academy of Medical Sciences, Tai’an, Shandong, China

**Keywords:** PDE8, cAMP, Alzheimer’s disease, cognition, microglia, neuroinflammation

## Abstract

**Background:**

Alzheimer’s disease (AD) is a neurodegenerative disease characterized by memory deficit and has emerged as a growing global health concern. Phosphodiesterase-8 (PDE8) is a cyclic adenosine monophosphate (cAMP)-specific hydrolase and its correlation with AD pathogenesis remains underexplored. Here, the effects and mechanisms of PF-04957325 (denoted as PF), a PDE8 inhibitor, were investigated in reversing AD both in vitro and in vivo.

**Methods:**

Briefly, BV2 cells were incubated with amyloid-β oligomers (AβO) to construct an AD cell model. Then, 2-month-old male C57BL/6J mice injected with AβO into the hippocampus and 10-month-old male amyloid precursor protein/presenilin-1 (APP/PS1) mice were used to construct AD animal models. Cells and mice were treated with PF to observe the effects of PDE8 on behavior and pathology related to AD. The Y-maze, novel object recognition (NOR), and Morris water maze (MWM) were performed to investigate cognitive function in mice. Western blot and immunofluorescence staining were used to identify the microglial activation state. Lastly, Western blot and ELISA were conducted to determine the levels of inflammatory factors and the proteins of PDE8/cAMP/CREB signaling.

**Results:**

PF-04957325 pretreatment reversed the conversation of proinflammatory microglia in BV2 cells induced by AβO, while also suppressing the levels of inflammatory factors, including interleukin-1β, interleukin-6, tumor necrosis factor-α, inducible nitric oxide synthase , and cyclooxygenase-2. In addition, AβO incubation upregulated the expression of PDE8 and concurrently downregulated that of brain-derived neurotrophic factor (BDNF), cAMP, p-PKA/PKA, and p-CREB/CREB in BV2 cells, all of which were reversed by PF. In vivo experiments evidenced impaired performance in the Y-maze, NOR, and MWM; these effects were reversed by PF. Similarly, PF treatment significantly attenuated microglia activation and the release of the inflammatory factors, and reversed the changes in the expression of BDNF and PDE8/cAMP/CREB signaling in AD mice. Finally, PF reduced the generation of Aβ_1-42_ by suppressing the expression of APP and PS1 in APP/PS1 mice.

**Conclusions:**

PF alleviated AD-like changes in behavior and pathology through various mechanisms, including attenuating microglia-mediated neuroinflammation, upregulating the expression of BDNF, restoring synaptic dysfunction, and inhibiting Aβ generation, which appear to be involved by PDE8/cAMP/CREB signaling. These results highlight the therapeutic potential of targeting PDE8 inhibition for AD treatment.

**Significance Statement**

Phosphodiesterase-8 (PDE8), similar to PDE4 and PDE7, is a cAMP-specific hydrolase, and the correlation with Alzheimer’s disease (AD) pathogenesis remains underexplored. Here, the effects and mechanisms of PF-04957325, a PDE8 inhibitor, were investigated in reversing AD both in vitro and in vivo. BV2 cells were incubated with amyloid-β oligomers (AβO) to construct an AD cell model. Then, 2-month-old male C57BL/6J mice injected with AβO into the hippocampus, and 10-month-old male amyloid precursor protein/presenilin-1 mice were used to construct AD animal models. PF alleviated AD-like changes in behavior and pathology through various mechanisms, including attenuating microglia-mediated neuroinflammation, upregulating the expression of BDNF, restoring synaptic dysfunction, and inhibiting Aβ generation, which appear to be involved by PDE8/cAMP/CREB signaling. These results highlight the therapeutic potential of targeting PDE8 inhibition for AD treatment, which holds significant implications for evaluating the biological role of PDE8 and developing targeted drugs.

## INTRODUCTION

Alzheimer’s disease (AD) is a chronic neurodegenerative disease characterized by progressive deterioration of cognitive capacity and behavioral impairment.^1^ With the growing aging population, the prevalence of AD steadily increases worldwide.^2^ At present, while drugs used in the clinical setting are effective in relieving the symptoms of AD, they do not cure or delay disease progression.^3^ To date, there are over 55 million individuals around the world suffering from AD and other dementias.^4^ In the absence of effective cures and preventive measures, this number is predicted to triple by 2050, imposing a significant emotional and economic burden on patients and their families.^5,6^ Therefore, there is a pressing need to identify novel drugs that can slow down or halt AD progression. At present, although beta-amyloid (Aβ) plaques and neurofibrillary tangles are considered pathological hallmarks of AD, the pathogenesis of AD remains elusive_._^7^ Inflammation has garnered extensive attention in recent years owing to its significant role in AD.^8^ Neuroinflammation, the inflammatory responses in the central nervous system (CNS) triggered by different types of injuries, is involved in the occurrence of numerous diseases.^9^ Previous studies have identified a vicious cycle wherein inflammation drives the synthesis of Aβ and abnormal tau phosphorylation. In turn, Aβ and phosphorylated Tau promote inflammation.^10^ This cyclical interaction presents a promising target for designing pharmacotherapies to mediate inflammation in AD.

Phosphodiesterases (PDEs), a superfamily of hydrolytic enzymes, degrade the second messengers cyclic adenosine monophosphate (cAMP) and cyclic guanosine monophosphate, which are crucial regulators in various physiological processes.^11^ Earlier studies described that cAMP exerts potent cognitive benefits by enhancing the protein kinase A (PKA)/cAMP response element-binding protein (CREB) signaling, which plays a pivotal role in memory consolidation.^12-14^ Meanwhile, cAMP has been recognized as an inducer of anti-inflammatory responses, with cAMP-dependent pathways being extensively targeted clinically for the treatment of inflammatory diseases.^15^ Consequently, inhibiting PDE activity and elevating cAMP levels may potentially exert AD-improving effects, potentially via the regulation of inflammatory pathways. Among PDE inhibitors, the most extensively studied are PDE4 inhibitors such as rolipram and roflumilast.^16-18^ Notably, PDE4 inhibitors are not typically used for the treatment of AD due to their side effects such as nausea and vomiting.^19^

PDE8, similar to PDE4, is a cAMP-specific hydrolase with a cAMP affinity 40-100 times greater than that of PDE4.^20^ Research enrolling AD patients reported that PDE8B, one of the 2 subtypes of PDE8, was the only isozyme with significantly elevated high levels in cerebrocortical areas and parts of the hippocampal formation at Braak stages III-VI.^21^ Meanwhile, animal research indicated that inactivation of the PDE8B gene can enhance learning and memory functions in mice.^22^ These findings indicate that PDE8 may participate in the pathogenesis of AD. Besides, targeting PDE8 has been reported to achieve therapeutic effects on diseases such as multiple sclerosis and asthma by reducing inflammatory lesions,^23^ indicating that inhibition of PDE8 can attenuate inflammation. However, it is not clear whether PDE8 inhibition improves AD and if inflammation is involved as the related mechanisms? In the present study, BV2 cells and mice were used to construct AD in vitro/in vivo models using Aβ oligomers (AβO) to explore the effect and mechanism of PDE8 inhibition in AD, which holds significant implications for evaluating the biological role of PDE8 and developing targeted drugs.

## MATERIALS AND METHODS

### Drug Preparation

Monomeric Aβ_1–42_ was purchased from GL Biochem, and AβO was prepared as previously described.^24^ Briefly, Aβ_1–42_ peptide was dissolved in dimethyl sulfoxide (DMSO) and then mixed with Ham’s/F12 medium (Procell) to obtain the stock solution. The solution was incubated at 4 °C overnight and centrifuged at 13,000 × *g* for 10 min at 4 °C. Then, the AβO was presented in the supernatant and stored at − 80 °C. For cell experiments, AβO stock solution was added to the cell culture medium to obtain a final concentration of 10 μM, making the final DMSO concentration of 0.5‰, and Ham’s/F12 medium containing 0.5‰ DMSO was used as vehicle. For animal experiments, AβO stock solution was dissolved in (phosphate buffer solution [PBS]) to obtain a final concentration of 0.4 μg/μL, and PBS containing 84% Ham’s/F12 medium and 0.44% DMSO was used as vehicle.

PF-04957325 (denoted as PF), as PDE8 inhibitor, was purchased from MCE. The IC50 values of PF on PDE8A and PDE8B were 0.7 and 0.4 nM, respectively,^23^ and more than 1.5 μM against all other PDE isoforms.^25^ Therefore, based on the results of our preliminary experiments, the concentrations of 150 nM, 300 nM, and 600 nM were selected for further experiments. PF-04957325 was dissolved in DMSO to obtain a stock solution and stored at − 80 °C. For cell experiments, the stock solution was added to the cell culture medium to obtain a final concentration of 150 nM, 300 nM, and 600 nM, and 0.6‰ DMSO was used as vehicle. For animal experiments, the stock solution was dissolved in PBS to obtain a final concentration of 0.1 mg/kg and 1 mg/kg, making the final DMSO concentration of 1.2%, which was used as vehicle.

### Cell Culture and Drug Treatment

Mouse microglial cell line BV2 cells were provided from KeyGEN Biotech Co., Ltd and were maintained in a 1640 medium supplemented (Gibco) with 10% fetal bovine serum (Cytiva) and 1% streptomycin and penicillin (Gibco). The cells were grown in a humidified incubator at 37 °C with 5% CO_2_. Cells at passages 3‑5 were utilized for subsequent experiments. BV2 cells were seeded into 6-well culture plates at 2 × 10^5^ cells/well mixed and cultured in an incubator for 24 h. BV2 cells were pretreated with PF (150 nM, 300 nM, or 600 nM) for 6h and then treated with AβO (10 μM) for 24 h. After that, the treated cells were collected for subsequent experiments. Each experiment was repeated 4 times, each time with 3 replicates.

### Experimental Animals

Two-month-old male C57BL/6J mice were obtained from Beijing HFK Bioscience Co. Ltd to establish AD model by microinjecting AβO into the hippocampus. Male APP^swe^/PS1^dE9^ (amyloid precursor protein/presenilin-1 [APP/PS1]) double transgenic mice and male wild-type (WT) mice in the same genetic background were also purchased from Beijing HFK Bioscience Co. Ltd and raised until 10 months old for experimentation. All mice were housed in a temperature- and light-controlled environment (23 °C, 45% humidity, 12-h light/12-h dark cycle) with free access to food and water in the specific pathogen free (SPF) animal facilities at the Institute of Pharmacology Shandong First Medical University. All experiments were performed in accordance with the protocols approved by the Laboratory Animals’ Ethic Committee of Shandong First Medical University. The experimental animal license was SYXK (Lu) 2017-0023.

### In Vivo Experiment Design

As previously described,^26^ AβO or its vehicle was delivered into the bilateral intrahippocampal CA1 area of C57BL/6J mice by stereotactic injection (0.8 μg/side, 0.5 μL/min). The following coordinates were used: anterior-posterior, –2.3 mm; lateral, ± 1.8 mm; and vertical, –2.0 mm. For all injections, the bregma was the reference point. The mice were administered by gavage with PF at the dose of 0.1 or 1 mg/kg or its vehicle for consecutive 21 days after 3 days of AβO stimulation. Similarly, 10-month-old male APP/PS1 mice and WT mice were also administered by gavage with PF at the dose of 0.1 or 1 mg/kg or its vehicle for consecutive 21 days. From the 15th to 21st day of administration, all mice were subjected to various behavioral tests in the following order: Y-maze, the novel object recognition (NOR), and the Morris water maze (MWM); all behavioral experiments were conducted 2 h after gavage administration.

### The Y-Maze Test

This was conducted in a Y-maze apparatus with 3 arms (40 cm long, 32 cm high, and 16 cm wide) that were intersected at 120°. Each mouse was placed in the central area to explore each arm. The number of entries into the arms and alterations were recorded for 10 min with the TopScan Package (Clever Sys Inc.). Spontaneous alternation was calculated according to the following formula: [(number of alternations)/ (total number of arm entries − 2)] × 100% as described in a previous paper.^27^ The apparatus was wiped and cleaned with 75% alcohol after each mouse.

### The NOR Test

This was conducted in a blue rectangular open field apparatus (60 × 60 × 60 cm) for 3 consecutive days as previously described.^27^ On the first day, the mice were individually allowed to freely explore the empty field for 10 min. On the second day, mice were individually placed in the arena to explore the 2 identical objects for 10 min. The last day, the mice were still allowed to freely explore the field for 10 min, but one of the 2 objects from yesterday was replaced by a novel object. The time spent on each object by the mice was recorded, and the recognition object index was calculated as a percentage of the time spent on the novel object over the total time spent exploring both objects. The tracking information was processed by the TopScan Package.

### The MWM Test

The MWM was conducted to examine spatial learning and memory in this study as described.^27^ In brief, the mission lasted for 6 days and consisted of 2 phases: the navigation trial (the first 5 days) and the probe trial (the sixth day). In the navigation trial, the mice were placed in the water to search for an escape platform within 60 s, and the escape latency was recorded for each mouse and each day. In the probe trial, the platform was removed and mice were placed from the opposite side of the previous platform quadrant to swim for 60 s. The time at which the mouse first arrived at the location of the escape platform, the times crossing the escape platform, the time in platform quadrant, and the total swimming distance were recorded for each mouse. The tracking information was processed by the TopScan Package.

### Brain Tissue Sampling

One hour after the last behavioral test (ie, the MWM), the mice were sacrificed under decapitation, and brains were collected. The hemispheres of 3 mice in each group were collected for pathological staining. The hippocampus of the rest mice was isolated from the hemispheres for Western blotting and ELISA assays; tissues were stored at −80 °C until analysis. For the immunofluorescence assay, the hemispheres of mice were fixed with 4% paraformaldehyde (PFA), then dehydrated, and embedded in paraffin.

### Western Blotting

The hippocampus from hemispheres and treated BV2 cells were homogenized, and protein extracts were prepared as previously described.^27^ The protein concentration was determined by the BCA Protein Quantification Kit (Solarbio). The extracts were separated by 6%–12% sodium dodecyl sulfate polyacrylamide gel electrophoresis according to the molecular weight of the target proteins and electro transferring to polyvinylidene fluoride (PVDF) membrane (Merck Millipore, IBFP0785C) The membranes were blocked in 5% skim milk and incubated in primary antibodies for 4 °C overnight with appropriate dilution listed in Table 1. Following washing, the membranes were incubated using a secondary antibody with the appropriate dilution listed in Table 1. The protein signals were visualized, and sensitometry analyses were conducted using the Image-Pro Plus software version 6.0 (Media Cybernetics Corp). The signal intensities were compensated by β-actin as internal controls.

**Table.1:** Related information for antibodies.

Antibody	Dilution ratio	Company	City and country	Cat No.
Rabbit PDE8A polyclonal antibody	1:1000	Abcam	Cambridge, MA, USA	ab109597
Rabbit PDE8B polyclonal antibody	1:500	Invitrogen	Waltham, MA, USA	PD8B-201AP
Rabbit CD206 polyclonal antibody	1:1000	Abcam	Cambridge, MA, USA	ab64693
Rabbit CD68 polyclonal antibody	1:1000	Proteintech	Wuhan, China	28058-1-AP
Rabbit PSD-95 monoclonal antibody	1:1000	Abcam	Cambridge, MA, USA	ab238135
Rabbit SYP monoclonal antibody	1:50 000	Abcam	Cambridge, MA, USA	ab32127
Rabbit iNOS polyclonal antibody	1:1000	Abcam	Cambridge, MA, USA	ab178945
Rabbit COX-2 monoclonal antibody	1:1000	Abcam	Cambridge, MA, USA	ab179800
Rabbit BDNF monoclonal antibody	1:1000	Abcam	Cambridge, MA, USA	ab108319
Rabbit Aβ_1-42_ monoclonal antibody	1:1000	Abcam	Cambridge, MA, USA	ab271968
Rabbit APP monoclonal antibody	1:1000	Abcam	Cambridge, MA, USA	ab201060
Rabbit PS1 monoclonal antibody	1:1000	Abcam	Cambridge, MA, USA	ab76083
Rabbit BACE1 polyclonal antibody	1:500	Proteintech	Wuhan, China	12807-1-AP
Rabbit PKA antibody	1:1000	CST	BeverlyMA, USA	4782S
Rabbit p-PKA antibody	1:1000	CST	Beverly, MA, USA	5661S
Rabbit CREB antibody	1:1000	CST	Beverly, MA, USA	9197S
Rabbit p-CREB antibody	1:1000	CST	Beverly, MA, USA	9198S
Mouse β-actin antibody	1:1500	Zhongshan Jinqiao Biotechnology Co Ltd	Beijing, China	TA-09
Horseradish enzyme-labeled rabbit anti-goat IgG (H + L)	1:3000	Zhongshan Jinqiao Biotechnology Co Ltd	Beijing, China	ZB-2306
Horseradish enzyme-labeled Goat anti-mouse IgG (H + L)	1:3000	Zhongshan Jinqiao Biotechnology Co Ltd	Beijing, China	ZB-2305

Abbreviations: Aβ1-42, Amyloid-β; APP, amyloid precursor protein; BDNF, brain-derived neurotrophic factor; COX-2, cyclooxygenase-2; CREB, cAMP response element-binding protein; iNOS, inducible nitric oxide synthase; p-CREB, phosphorylated cAMP response element-binding protein; PDE8A, phosphodiesterase-8; PDE8B, phosphodiesterase-8; PKA, protein kinase A; p-PKA, phosphorylated protein kinase A; PS1, presenilin-1; PSD-95, postsynaptic density-95; SYP, synaptophysin.

### Immunofluorescence Assay

Cellular immunofluorescence was performed as described in a previous study.^27^ Briefly, cells were sequentially fixed with 4% PFA, permeabilized with 0.2% Triton X, blocked with 5% bovine serum albumin (BSA) for 1 h, and incubated with corresponding primary antibodies targeting CD68 (1:200, rabbit, 28058-AP, Proteintech) or targeting CD206 (1:200, rabbit, ab64693, Abcam) at 4 °C overnight. Next, they were incubated with Alexa Flour-conjugated secondary antibodies for 1 h at room temperature (RT) and subsequently mounted in a medium containing 6-diamidino-2-phenylindole (DAPI, 1:2000, G1012, ServiceBio) for 15 min at RT. Brain slices with a thickness of 5 μm underwent immunofluorescent staining as outlined in a previous study.^26^

Briefly, brain sections were dewaxed, and antigen retrieval was performed with citric acid antigen retrieval (pH 6.0) buffer in a microwave oven for 15 min, permeabilized with 0.5% Triton X-100, then blocked with 5% BSA for 1 h at RT, and stained with primary antibodies targeting CD68 (1:200, rabbit, 28058-1-AP, Proteintech) or targeting CD206 (1:200, rabbit, ab64693, Abcam) at 4 °C overnight. After being washed 3 times with PBS, the sections were incubated in anti-rabbit secondary antibodies (1:2000, SGB23303, ServiceBio) at RT for 50 min, and then horseradish peroxidase was permanently fluorescence-labeled in secondary antibody by TYR-CY3 (1:500, G1233, Servicebio) at RT for 10 min. Then, antigen retrieval was performed again with a citric acid antigen retrieval buffer (pH 6.0) for 20 min. After that, brain sections were blocked again with 5% BSA for 30 min at RT, stained with primary antibodies targeting IBA-1(1:200, rabbit, GB113502, ServiceBio) at 4 °C overnight, and incubated in anti-rabbit secondary antibodies (1:2000, SGB23303, ServiceBio) at RT for 50 min. Horseradish peroxidase was permanently fluorescence-labeled in a secondary antibody by TYR488 (1:500, G1231, ServiceBio) at RT for 10 min. Nuclei were stained with DAPI for 3 min. Images were captured under a fluorescence microscope (NIKON ECLIPSE C1, Japan). Fluorescent intensity was quantified using Image-Pro Plus software version 6.0 (Media Cybernetics Corp).

### ELISA

The level of tumor necrosis factor-α (TNF-α), interleukin-1β (IL-1β), interleukin-6 (IL-6), and cAMP were determined using Mouse Quantikine ELISA kits purchased from Shanghai enzyme-linked Biotechnology Co., Ltd according to the manufacturer’s instruction in BV2 cells or mouse hippocampus. Both the entire hippocampus from hemispheres and collected cells were homogenized with ice-cold PBS containing 1% phenyl methane sulfonyl fluoride and centrifuged at 5000 × *g* for 10 min and at 1000 × *g* for 20 min, respectively, then the supernatant was collected for Elisa analysis. Colorimetric reaction was conducted, and absorbance at 450 nm was recorded with a multifunctional microplate reader (Tecan). The BCA method was used to measure the protein concentrations with the Enhanced BCA Protein Assay Kit (Solarbio) according to the manufacturer’s instructions.

### Statistical Analyses

Statistical analyses were performed by Prism 8 (GraphPad Software). Quantitative data were expressed as mean ± SEM and analyzed using 1- and 2-way ANOVA followed by post hoc comparison using Tukey’s test. The difference was considered to be statistically significant when *P *< .05.

## RESULTS

### PF Reduced AβO-Induced Microglia Activation and Directed Microglia Polarization

**Figure 1.**
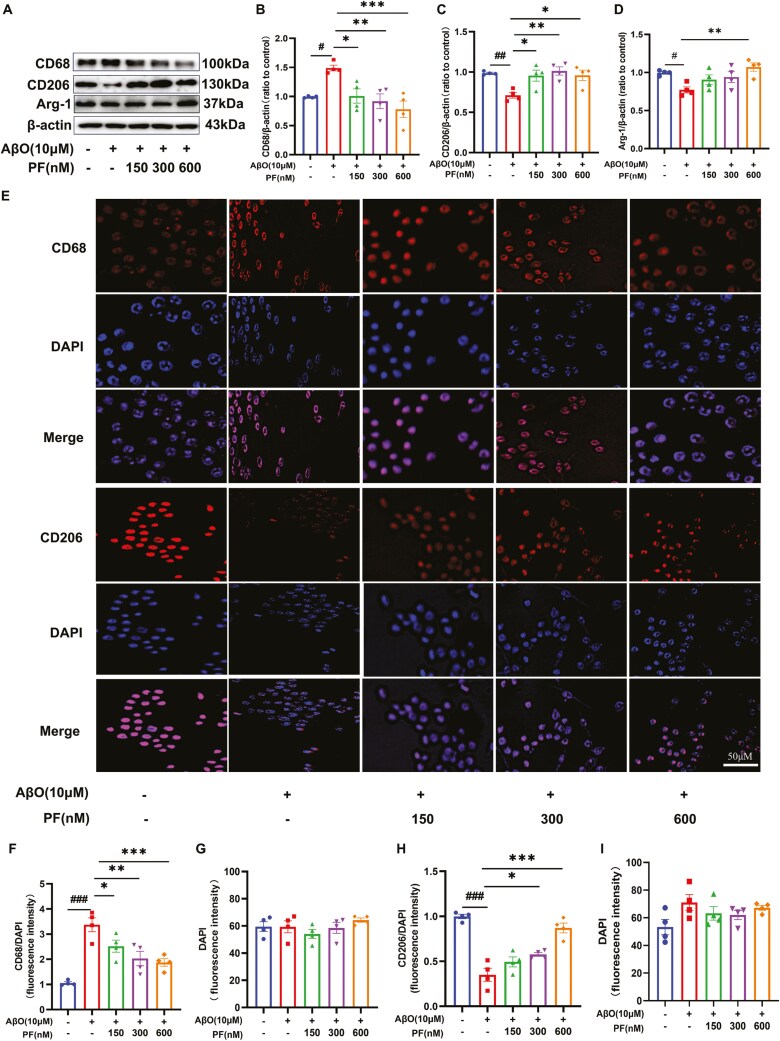
**PF reduced AβO-induced microglia activation and directed microglia polarization.** (A) Representative images by Western blotting showing the expression of CD68, CD206, and Arg-1 by PF pretreatment in AβO-exposed BV2 microglia. (B-D) Quantitative analysis of CD68, CD206, and Arg-1, normalized by β-actin to Vehicle. (E) Representative images by immunofluorescence showing the change of CD68 and CD206 by PF pretreatment in AβO-exposed BV2 microglia. (F-I) The quantification of CD68 and DAPI, CD206, and DAPI fluorescence intensity. CD68 and CD206: red, DAPI: blue. The values shown are means ± SEM, ^#^*P <* .05, ^*##*^*P <* .01, ^###^*P <* .001vs Vehicle; * *P <* .05, ** *P <* .01, *** *P <* .001vs AβO with vehicle; *n* = 4. AβO, amyloid-β oligomers; DAPI, 6-diamidino-2-phenylindole; PF, PF-04957325.

Microglia were classified into proinflammatory microglia and anti-inflammatory microglia, and activated microglia are one of the essential neurotoxic mediators of neuroinflammation.^28^To investigate the effect of PF on BV2 microglial activation induced by AβO, the expression of microglial activation markers was evaluated via Western blotting and immunofluorescence assays. The results revealed that the expression of proinflammatory microglia biomarker CD68 was upregulated, while that of the anti-inflammatory microglia biomarker CD206 and Arg-1 was significantly downregulated in AβO-exposed BV2 cells (Figure 1A-D; *P <* .05). Moreover, PF pretreatment dose-dependently reversed the expression of CD68, CD206, and Arg-1 induced by AβO in BV2 cells (Figure 1A-D; *P <* .05). As anticipated, the results of the immunostaining assay were in line with those of Western blotting analysis (Figure 1E-I).

### PF Attenuated Expression of Proinflammatory Mediators in AβO-Exposed BV2 Cells

**Figure 2.**
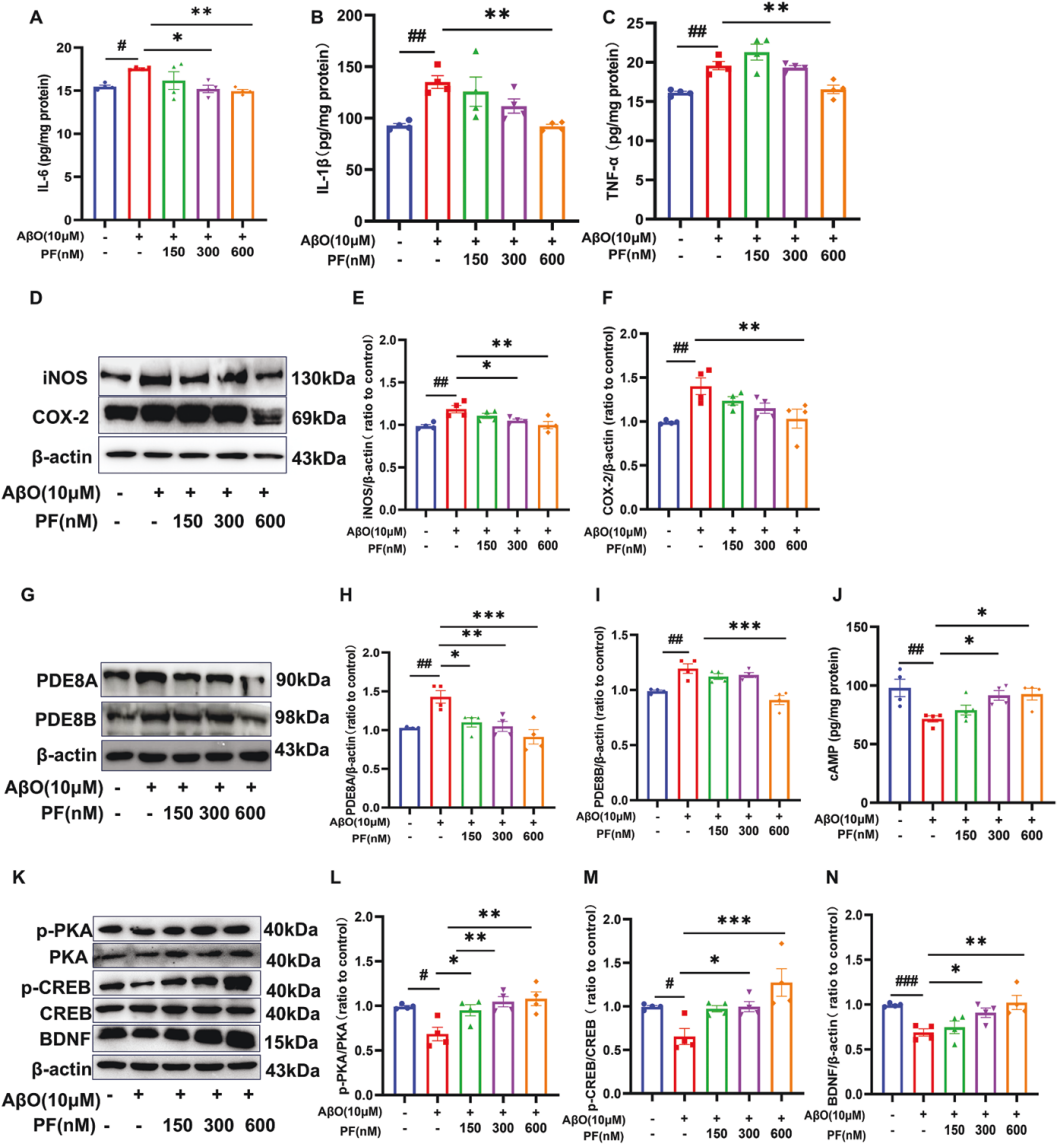
**PF attenuated the expression of pro-inflammatory mediators and regulated the PDE8/cAMP/CREB signaling pathway in AβO-exposed BV2 cells.** (A-C) The effect PF on IL-6, IL-1β, and TNF-α level determined by ELISA assay in AβO-exposed BV2 microglia. (D) Representative images by Western blotting showing the expression of iNOS and COX-2 by PF pretreatment in AβO-exposed BV2 microglia. (E-F) Quantitative analysis of iNOS and COX-2. (G) Representative images by Western blotting showing the expression of PDE8A and PDE8B by PF pretreatment in AβO-exposed BV2 microglia. (H-I) Quantitative analysis of PDE8A and PDE8B, normalized by β-actin to Vehicle. (J) The effect of PF on cAMP level determined by ELISA assay in AβO-exposed BV2 microglia. (K) Representative images by Western blot showing the expression of p-PKA, PKA, p-CREB, CREB, and BDNF by PF pretreatment in AβO-exposed BV2 microglia. (L-N) Quantitative analysis of p-PKA, PKA, p-CREB, CREB, and BDNF by PF pretreatment in AβO-exposed BV2 microglia The values shown are means ± SEM, ^#^*P <* .05, ^*##*^*P <* .01, ^###^*P <* .001 vs Vehicle; * *P <* .05, ** *P <* .01, ***P < .001 vs AβO with vehicle; *n* = 4. AβO, amyloid-β oligomers; AD, Alzheimer’s disease; cAMP, cyclic adenosine monophosphate; BDNF, brain-derived neurotrophic factor; COX-2, cyclooxygenase-2; CREB, cAMP response element-binding protein; IL-1β, interleukin-1 beta; IL-6, interleukin-6; iNOS, inducible nitric oxide synthase; p-CREB, phosphorylated cAMP response element-binding protein; PDE8, phosphodiesterase-8; PF, PF-04957325; PKA, protein kinase A; p-PKA, phosphorylated protein kinase A.

To determine the effects of PF on the levels of AβO-induced inflammatory mediators, the expression levels of IL-1β, IL-6, TNF-α, inducible nitric oxide synthase (iNOS), and cyclooxygenase-2 (COX-2) were assessed using ELISA and Western blotting analysis. The results unveiled that the levels of proinflammatory cytokines were significantly increased following AβO stimulation, as evidenced by the increased production of IL-6 (Figure 2A; *P <* .05), IL-1β (Figure 2B; *P <* .01), TNF-α, iNOS, and COX-2 (Figure 2C-F; *P <* .01). However, while pretreatment with 150 nM PF decreased the levels of some proinflammatory cytokines, their levels were comparable to those in AβO-treated BV2 cells. At the same time, pretreatment with 300 nM PF decreased the expression levels of IL-6 and iNOS (Figure 2A, D, and E; *P <* .05), whereas pretreatment with 600 nM PF reversed all the increases in the levels of inflammatory mediators (Figure 2A-F; *P <* .01).

### PF Regulated the PDE8/cAMP/CREB Signaling Pathway in AβO-Exposed BV2 Cells

To further explore the mechanism by which PF attenuates inflammation in AβO-treated BV2 cells, the expression of PDE/cAMP/CREB signaling pathway-related proteins was detected using Western blotting analysis and ELISA. The results demonstrated that the expression levels of PDE8A and PDE8B were significantly higher in AβO-stimulated BV2 cells compared to control cells (Figure 2G-I; *P <* .01). However, increased expression of PDE8A was dose-dependently reversed by PF pretreatment, while the high expression of PDE8B was only reversed by PF at 600 nM (Figure 2I; *P <* .001). Additionally, cAMP levels, the p-PKA/PKA ratio, and the p-CREB/CREB ratio were significantly lower in AβO-exposed BV2 cells compared to control cells (Figure 2J-N; *P <* .05). Interestingly, the downregulation of cAMP and the p-CREB/CREB ratio was reversed by pretreatment with 300 and 600 nM PF (Figure 2J and M; *P <* .05), while the p-PKA/PKA ratio was reversed by PF pretreatment in a dose-dependent manner (Figure 2K and L; *P <* .05). Furthermore, the results revealed that the expression level of brain-derived neurotrophic factor (BDNF) was significantly lower in AβO-exposed BV2 cells compared to control cells, which was reversed by PF at 300 and 600 nM (Figure 2N; *P <* .01).

### PF Attenuated Cognitive Impairment in AβO-Treated Mice and APP/PS1 Mice

**Figure 3.**
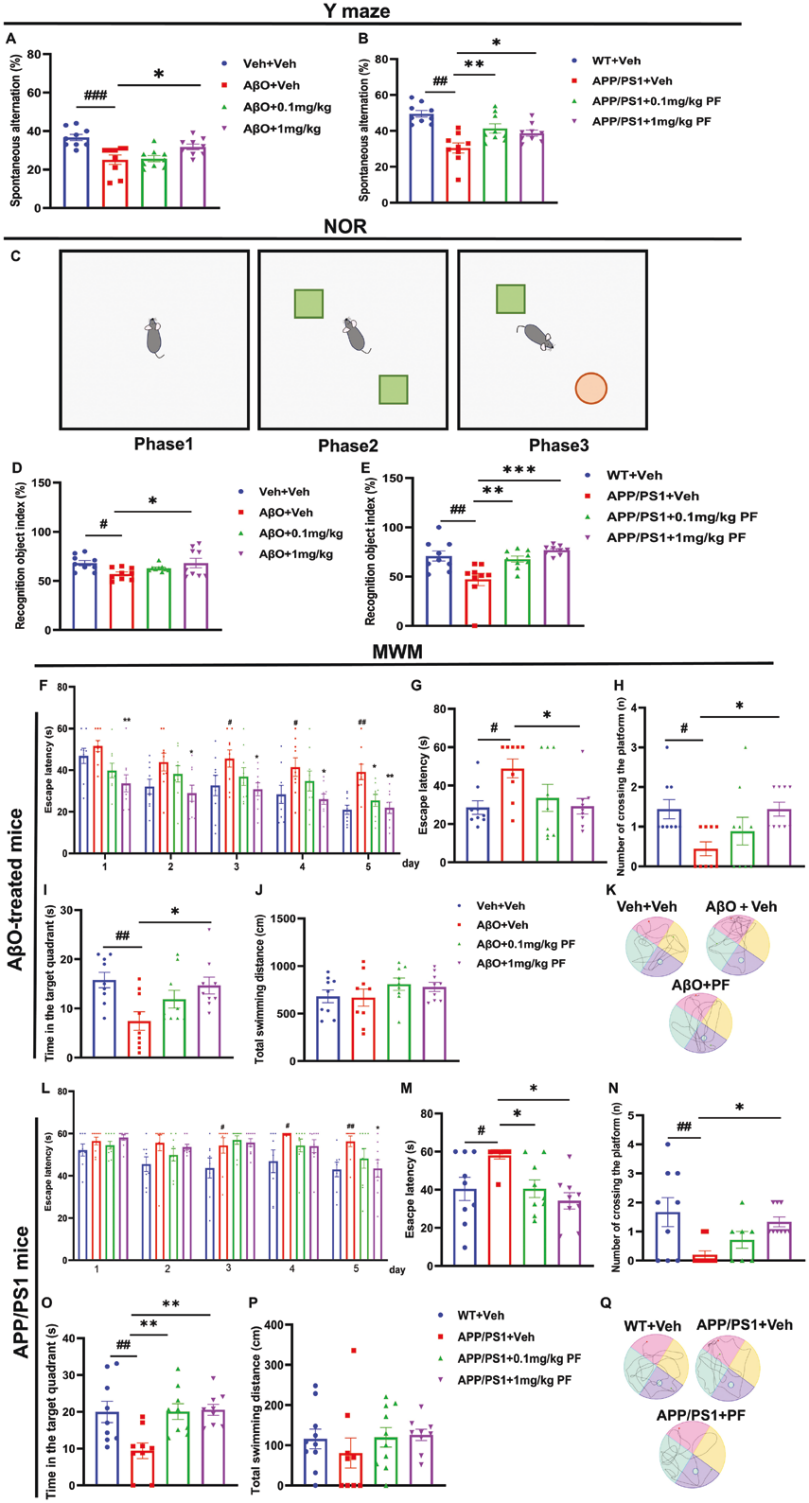
**PF improved cognitive impairment in AβO-induced AD model mice and APP/PS1.** In the Y-maze, the spontaneous alternation was recorded (A-B). (C) Representative occupancy plots of animals’ exploration of the novel object (red) and the familiar object (green) in the NOR. (D-E) In NOR, the recognition object index was calculated as the percentage of time exploring the novel object over the total time exploring both objects. In the acquisition trial of MWM, all mice were tested for 5 consecutive days to locate the hidden platform, and the escape latency (F) was recorded. During the probe trial, the platform was removed, and the escape latency, which is the time entering the previous platform area (target quadrant) for the time (G) number of crossing the platform (H) time in the target platform quadrant (I) and the swimming distance (J) and swimming paths (K) in mice were recorded. In the acquisition trial of MWM, all mice were tested for 5 consecutive days to locate the hidden platform, and the escape latency (L) was recorded. During the probe trial, the platform was removed, and the escape latency, which is the time entering the previous platform area (target quadrant) for the time (M), the number of crossing the platform (N), time in the target platform quadrant (O), and the swimming distance (P) and swimming paths (Q) in mice were recorded. The values shown are means ± SEM, ^#^*P <* .05, ^##^*P <* .01, ^###^*P* < .001vs Vehicle or WT with vehicle. *P < .05, **P < .01, ***P < .001 vs AβO with vehicle or APP/PS1 with vehicle; *n* = 9. AβO, amyloid-β oligomers; AD, Alzheimer’s disease; APP, amyloid precursor protein; MWM, Morriz water maze; NOR, novel object recognition; PF, PF-04957325; PS1, presenilin-1; WT, wild type.

To further investigate the effect of PDE8 inhibition on AD, an AβO-injected AD-like cognitive deficit mouse model and APP/PS1 mice were used. The Y-maze, NOR, and MWM tests were used to evaluate learning and memory abilities. In the Y-maze, the spontaneous alternation was decreased in AβO-injected mice compared to control mice. This effect was reversed by PF at 1 mg/kg (Figure 3A; *P <* .05). Similarly, the number of entries into the arms and the spontaneous alternation in APP/PS1 mice were lower compared to WT mice; this was reversed by PF in a dose-dependent manner (Figure 3B; *P* < .05). In the NOR, as shown in the graph about the object preference of mice (Figure 3C), the recognition index was lower in AβO-injected mice and APP/PS1 mice compared to control mice and WT mice, respectively; these were reversed by PF at 1 mg/kg orally (Figure 3D and E; *P <* .05).

Furthermore, during the acquisition trail of the MWM, the escape latency recorded in AβO-stimulated mice was significantly prolonged compared with the control mice from day 3, while PF at 0.1 mg/kg only shortened the escape latency in AβO-stimulated mice on the fifth day, and PF at 1 mg/kg reduced the escape latency from day 1 (Figure 3F; *P* < .05). During the probe trial, the escape latency was significantly prolonged in AβO-stimulated mice compared to the control mice, and the number of crossings into the target quadrant and the percent of time in the platform quadrant was less; all of which were reversed by PF at 1 mg/kg (Figure 3G-I; *P* < .05). Similarly, the escape latency recorded during the acquisition trial was significantly prolonged in APP/PS1 mice compared to WT mice from day 3 and that was reserved by PF at 1 mg/kg on day 5 (Figure 3L; *P* < .05). During the probe trial, PF dose-dependently reserved the prolong of escape latency and the reduction of the percent of time in the platform quadrant in APP/PS1 mice, while only 1 mg/kg PF elevated the number of crossings into the target quadrant in APP/PS1 mice (Figure 3M-O; *P* < .05). There was no significant difference in swimming distance for all mice in the 2 experiments (Figure 3J and P; *P* > .05). Moreover, the characteristic swimming paths of different groups were illustrated in the probe test on the sixth day as shown in Figure 3K and Q.

### PF Moderated Microglia Activation and Proinflammatory Cytokine Expression in AβO-Treated Mice and APP/PS1 Mice

**Figure 4.**
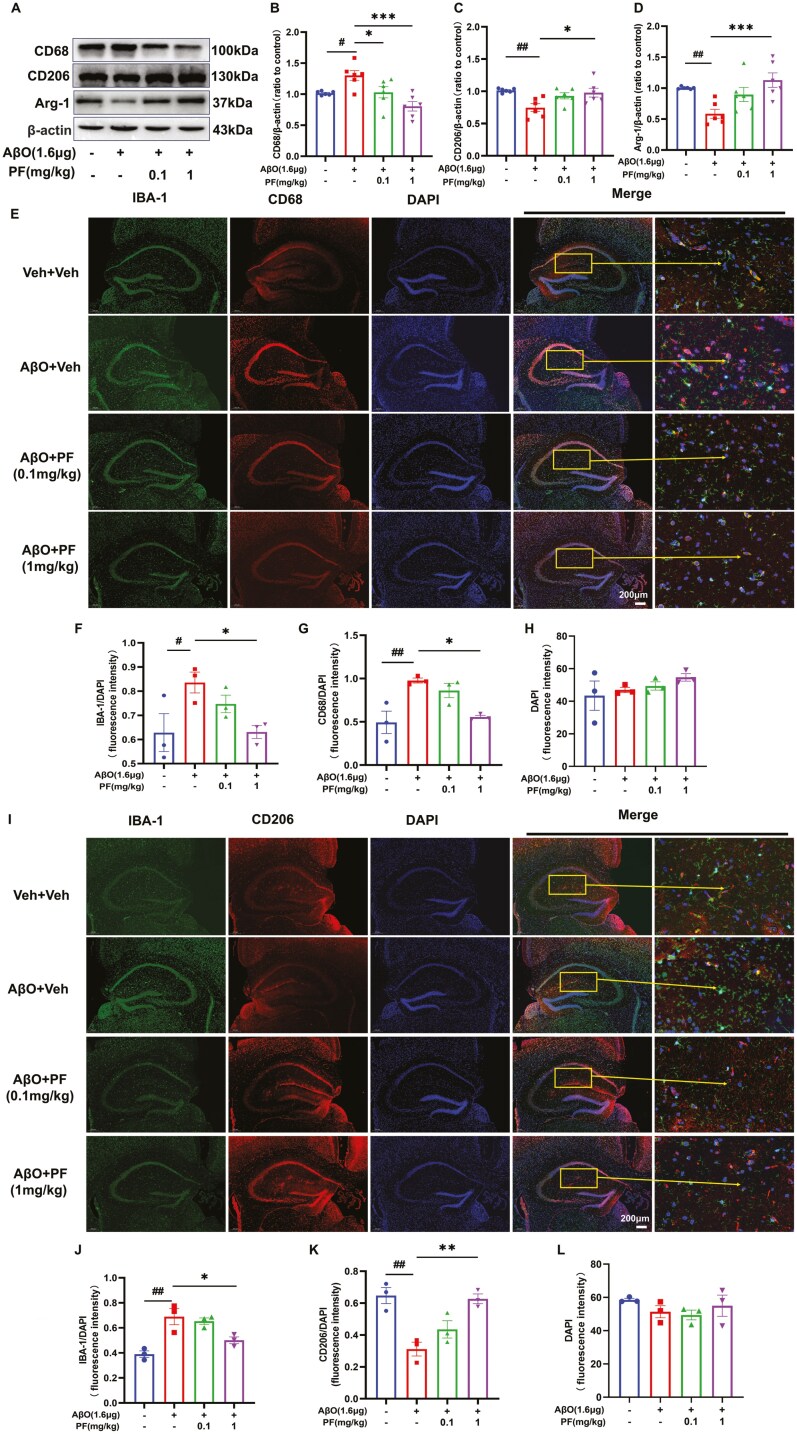
**PF moderated microglia activation in AβO-induced AD model mice.** (A) Representative images by Western blotting showing the expression of CD68, CD206, and Arg-1 by PF pretreatment in AβO-induced AD model mice. (B-D) Quantitative analysis of CD68, Arg-1, and CD206, normalized by β-actin to control. (E) Representative images of IBA-1 and CD68 by immunofluorescence staining in the hippocampus. (F) The quantification of IBA-1 in the hippocampus. (G) The quantification of CD68 in the hippocampus (H) The fluorescence intensity of DAPI in the hippocampus. (I) Representative images of IBA-1 and CD206 by immunofluorescence staining in the hippocampus. (J) The quantification of IBA-1 in the hippocampus. (K) The quantification of CD206 in the hippocampus. (L)The quantification of IBA-1 in the hippocampus. IBA-1: green, CD68 and CD206: red, DAPI: blue. The values shown are means ± SEM, ^*#*^*P <* .05, ^*##*^*P <* .01 vs vehicle; *P < .05, **P < .01, ***P < .001 vs AβO with vehicle; for Western blotting and ELISA, *n* = 6, for immunofluorescence staining, *n* = 3. AβO, amyloid-β oligomers; AD, Alzheimer’s disease; DAPI, 6-diamidino-2-phenylindole; PF, PF-04957325.

**Figure 5.**
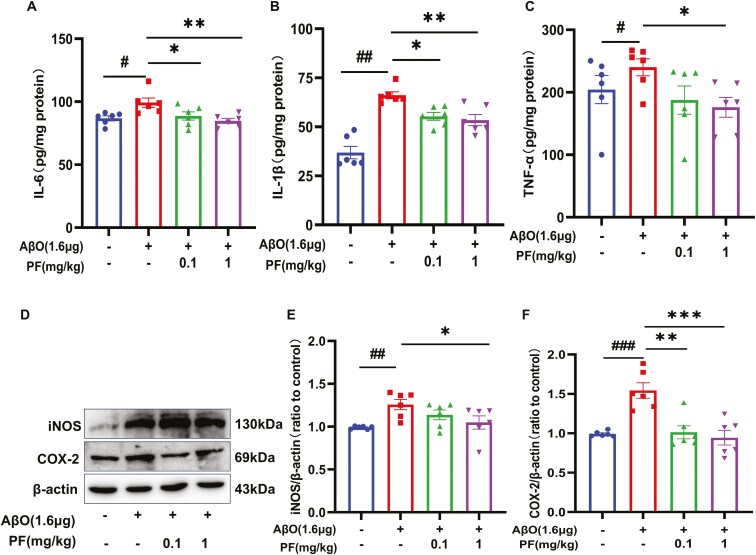
**PF moderated proinflammatory cytokine expression in AβO-induced AD model mice.** (A-C) The level of IL-6, IL-1β, and TNF-α by PF pretreatment in AβO-induced AD model mice was determined by ELISA assay. (D) Representative images by Western blotting showing the expression of iNOS and COX-2 by PF pretreatment in AβO-induced AD model mice. (E-F) Quantitative analysis of iNOS and COX-2, normalized by β-actin to vehicle. The values shown are means ± SEM, ^#^*P* < .05, ^##^*P* < .01, ^###^*P* < .001 vs vehicle; *P < .05, **P < .01, ***P < .001 vs AβO with vehicle; *n* = 6. AβO, amyloid-β oligomers; AD, Alzheimer’s disease; COX-2, cyclooxygenase-2; iNOS, inducible nitric oxide synthase ; PF, PF-04957325.

In order to explore the effect of PF on microglial polarization in vivo, the expression of CD68 and CD206 of the hippocampus in mice was detected by Western blotting analysis and immunofluorescence staining. The results revealed that the expression of CD68 significantly increased, and the expression of CD206 and Arg-1 obviously decreased in AβO-induced AD model mice with respect to control mice; PF treatment reversed the expression of CD206 and Arg-1 with a significant effect at the concentration of 1 mg/kg and the expression of CD68 in a concentration-dependent manner in AβO-exposed mice (Figure 4A-D; *P <* .05). Similarly, the immunofluorescence staining results showed that the intensity of IBA-1 and CD68 was obviously elevated in mice after AβO treatment, while the density of CD206 decreased significantly; these changes were reversed by 1 mg/kg PF treatment (Figure 4E-L; *P* < .05). Furthermore, the release of inflammatory factors, such as IL-6, IL-1β, TNF-α, iNOS, and COX-2, was increased in the AβO-induced AD model mice with respect to the controls mice (Figure5A-F, *P* < .05); the expression levels of IL-1β, IL-6, and COX-2 were downregulated in AβO-induced AD model mice treated with 0.1 mg/kg and 1 mg/kg PF (Figure 5A, B, D, and F, *P* < .05); the expression levels of TNF-α and iNOS were downregulated only in AβO-induced AD model mice treated with 1 mg/kg PF (Figure 5C-E; *P* < .05).

**Figure 6.**
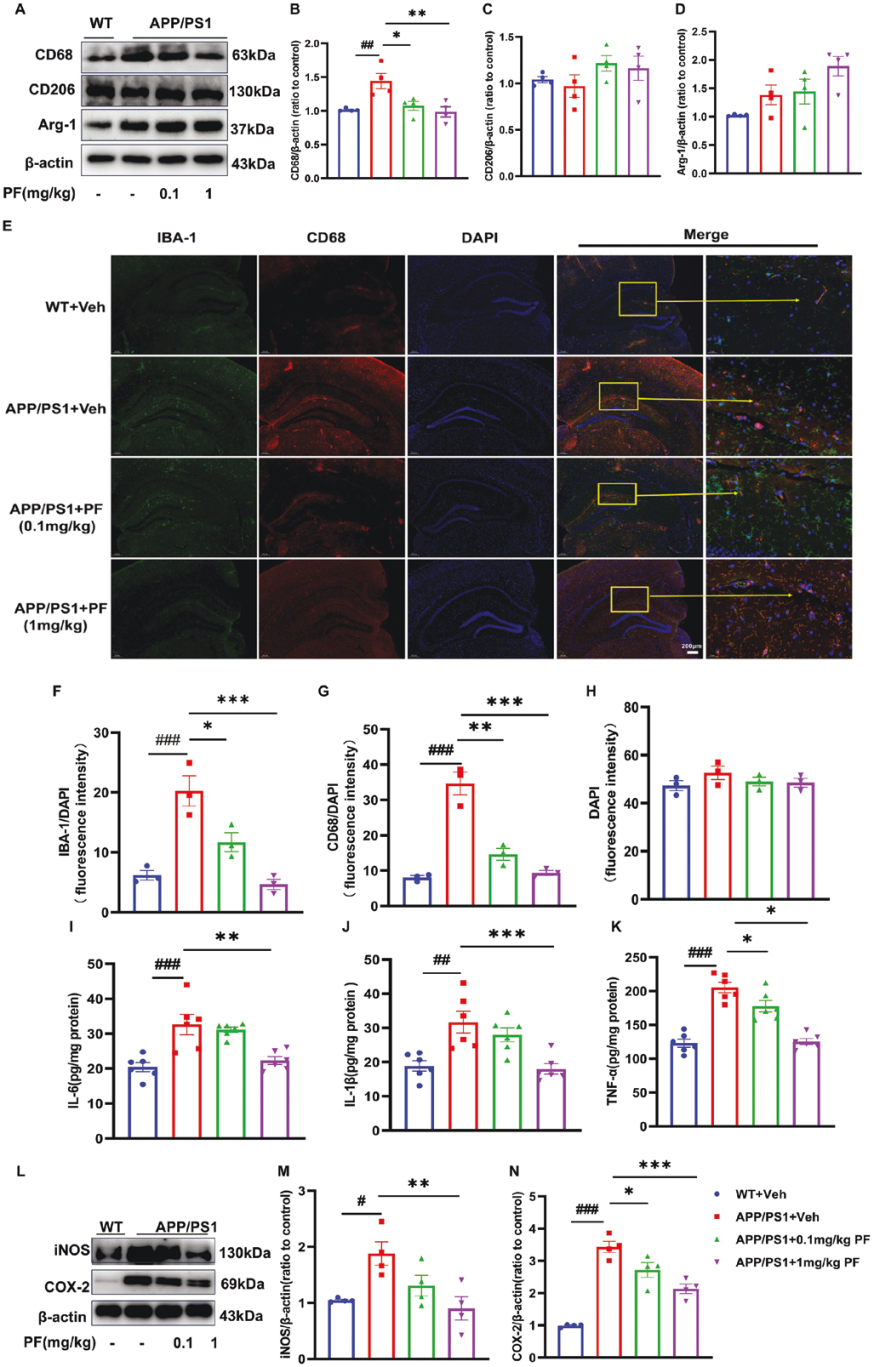
**PF moderated microglia activation and proinflammatory cytokine expression in APP/PS1 mice.** (A) Representative images by Western blotting showing the expression of CD68, CD206, and Arg-1 by PF pretreatment in APP/PS1 mice. (B-D) Quantitative analysis of CD68, CD206, and Arg-1, normalized by β-actin to WT with vehicle. (E) Representative images of IBA-1 and CD68 by immunofluorescence staining in the hippocampus. (F) The quantification of IBA-1 in the hippocampus (G) The quantification of CD68 in the hippocampus. (H) The quantification of DAPI in the hippocampus.(I-K) The level of IL-6, IL-1β, and TNF-α by PF pretreatment in APP/PS1 mice was determined by ELISA assay. (K) Representative images by Western blotting showing the expression of iNOS and COX-2 normalized by β-actin to WT with vehicle. (L-N) Quantitative analysis of iNOS and COX-2, normalized by β-actin to WT with vehicle. The values shown are means ± SEM, ^*#*^*P <* .05, ^*##*^*P <* .01, ^###^*P <* .001 vs Vehicle; *P < .05, **P < .01, ***P < .001 vs AβO plus vehicle vs AβO plus vehicle; ^#^*P <* .05, ^##^*P <* .01, ^###^*P <* .001vs WT with vehicle. *P < .05, **P < .01, ***P < .001 vs APP/PS1 with vehicle; for Western blotting and ELISA assay, *n* = 4, for immunofluorescence staining, *n* = 3. AβO, amyloid-β oligomers; APP, amyloid precursor protein; COX-2, cyclooxygenase-2; DAPI, 6-diamidino-2-phenylindole; IL-1β, interleukin-1 beta; IL-6, interleukin 6; iNOS, inducible nitric oxide synthase ; PF, PF-04957325; PS1, presenilin-1; TNF-α, tumor necrosis factor-alpha ; WT, wild type.

In APP/PS1 mice, indicators related to microglia polarization and neuroinflammation also were evaluated to expose the role of PF in the progression of AD. Western blotting results showed that the expression of CD68 was increased in the hippocampus of APP/PS1 mice (Figure 6A and B; *P* < .01), while there was no significant difference for CD206 and Arg-1 expression in the hippocampus of all mice (Figure 6A, C, and D). Meanwhile, the immunofluorescence staining results showed that the intensity of IBA-1 and CD68 was obviously elevated in APP/PS1 mice compared to WT mice, and PF dose-dependently inhibited the increase in APP/PS1 mice (Figure 6E-H; *P *< .01). Moreover, the expression of proinflammatory factors such as iNOS, COX-2, IL-6, IL-1β, and TNF-α was apparently elevated in the hippocampus of APP/PS1 mice compared to WT mice (Figure 6I-N; *P *< .05); all of these changes were reserved by PF at 1 mg/kg (Figure 6I-N; *P *< .05), while the expression of TNF-α and COX-2 in APP/PS1 mice could also be reversed by 0.1 mg/kg PF (Figure 6K, L, and N; *P* < .05).

### PF Regulated the PDE8/cAMP/CREB Signaling Pathway and Synapse-Associated Proteins in AβO-Treated Mice and APP/PS1 Mice

**Figure 7.**
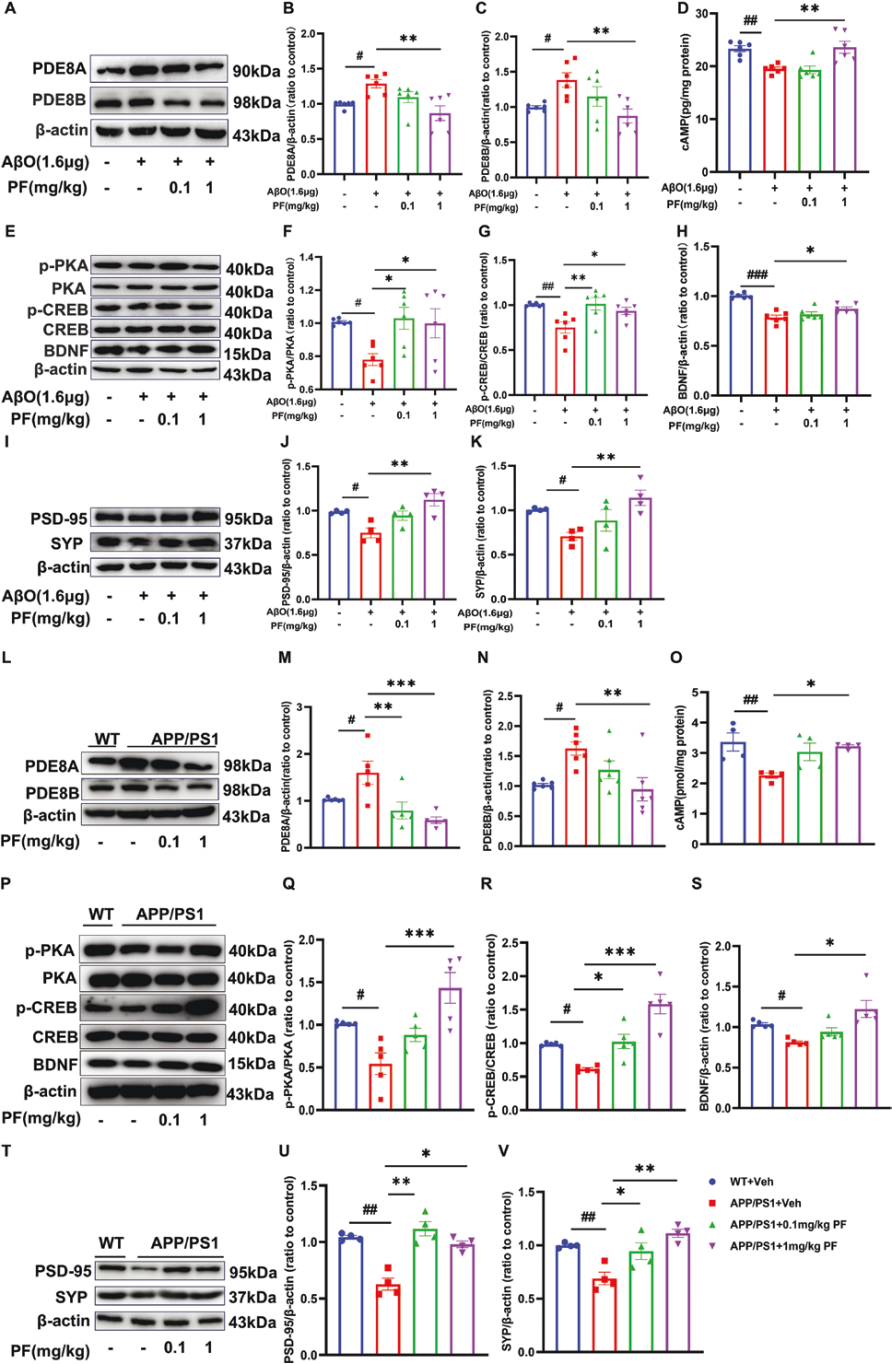
**PF attenuated proinflammatory cytokine expression, and synapse-associated proteins modulated PDE8/cAMP/CREB pathway in AβO-induced AD model mice and APP/PS1 mice.** (A) Representative images by Western blotting showing the expression of PDE8A and PDE8B by PF pretreatment in AβO-induced AD model mice. (B-C) Quantitative analysis of PDE8A and PDE8B, normalized by β-actin to AβO plus vehicle group. (D) The level of cAMP by PF pretreatment in AβO-induced AD model mice was determined by ELISA assay. (E) Representative images by Western blotting showing the expression of p-PKA, PKA, p-CREB, CREB, and BDNF by PF pretreatment in AβO-induced AD model mice. (F-H) Quantitative ratio of p-PKA/PKA, the ratio of p-CREB/CREB, and BDNF normalized by β-actin to AβO vehicle. (I) Representative images by Western blotting showing the expression of PSD-95 and SYP by PF pretreatment in AβO-induced mice. (J-K) Quantitative analysis of PSD-95 and SYP, normalized by β-actin to WT with vehicle. (L) Representative images by Western blotting showing the expression of PDE8A and PDE8B by PF pretreatment in APP/PS1 mice. (M-N) Quantitative analysis of PDE8A and PDE8B, normalized by β-actin to WT group. (O) The level of cAMP by PF pretreatment in APP/PS1 mice was determined by ELISA assay. (P) Representative images by Western blot showing the expression of p-PKA, PKA, p-CREB, CREB, and BDNF by PF pretreatment in APP/PS1 mice. (Q-S) Quantitative analysis of the ratio of p-PKA/PKA, the ratio of p-CREB/CREB, and BDNF normalized by β-actin to WT with vehicle. (T) Representative images by Western blotting showing the expression of PSD-95 and SYP by PF pretreatment in APP/PS1 mice. (U-V) Quantitative analysis of PSD-95 and SYP, normalized by β-actin to WT with vehicle. The values shown are means ± SEM, ^*#*^*P <* .05, ^*##*^*P <* .01, ^###^*P <* .001vs Vehicle or WT with vehicle; *P < .05, **P < .01, ***P < .001 vs AβO with vehicle or APP/PS1 with vehicle; *n* = 4-6. AβO, amyloid-β oligomers; AD, Alzheimer’s disease; APP, amyloid precursor protein; cAMP, cyclic adenosine monophosphate; BDNF, brain-derived neurotrophic factor; CREB, cAMP response element-binding protein; PF, PF-04957325; p-CREB, phosphorylated cAMP response element-binding protein; PKA, protein kinase A; p-PKA, phosphorylated protein kinase A; PS1, presenilin-1; WT, wild type.

In order to reveal the mechanism of PF on inflammation induced by AβO injection in mice hippocampus and APP/PS1 mice, the expression of PDE8/cAMP/CREB pathway-related proteins was detected through Western blotting and ELISA analysis. The results showed that the expression of PDE8A and PDE8B was indeed induced after AβO injection into the hippocampus of mice (Figure 7A-C, *P *< .05), accompanied by a significant decrease in downstream molecule cAMP, p-PKA/PKA ration, and p-CREB/CREB ration; all these changes were reversed by PF at 1 mg/kg orally (Figure 7D-G; *P *< .05). Furthermore, the expression levels of BDNF were obviously decreased in AβO-induced AD model mice and APP/PS1 mice compared to control groups; this was reversed by 1 mg/kg PF treatment (Figure 7H and S; *P <* .05). There is growing evidence that synaptic dysfunction and degeneration contribute to the deterioration of memory performance, and that BDNF plays an important role in maintaining synaptic plasticity in learning and memory^.29,30^ Therefore, the expression of SYP, PSD-95, and BDNF was deleted by Western blotting to explore the effect of PF on a synaptic disorder. The results indicated that the expression of SYP, PSD-95, and BDNF was significantly declined in the hippocampus of AβO-treated mice and APP/PS1 mice compared to control groups (Figure 7I-K and T-V, *P* < .05); all these were reversed by 1 mg/kg PF treatment (Figure 7H, J, K, S, U, and V; *P* < .05), while the expression of SYP and PSD-95 in hippocampus of APP/PS1 mice was reversed by 0.1 mg/kg PF treatment (Figure 7U-V, *P* < .05).

### PF Reduced the Production of Aβ_1-42_ in Hippocampus of APP/PS1 Mice

**Figure 8.**
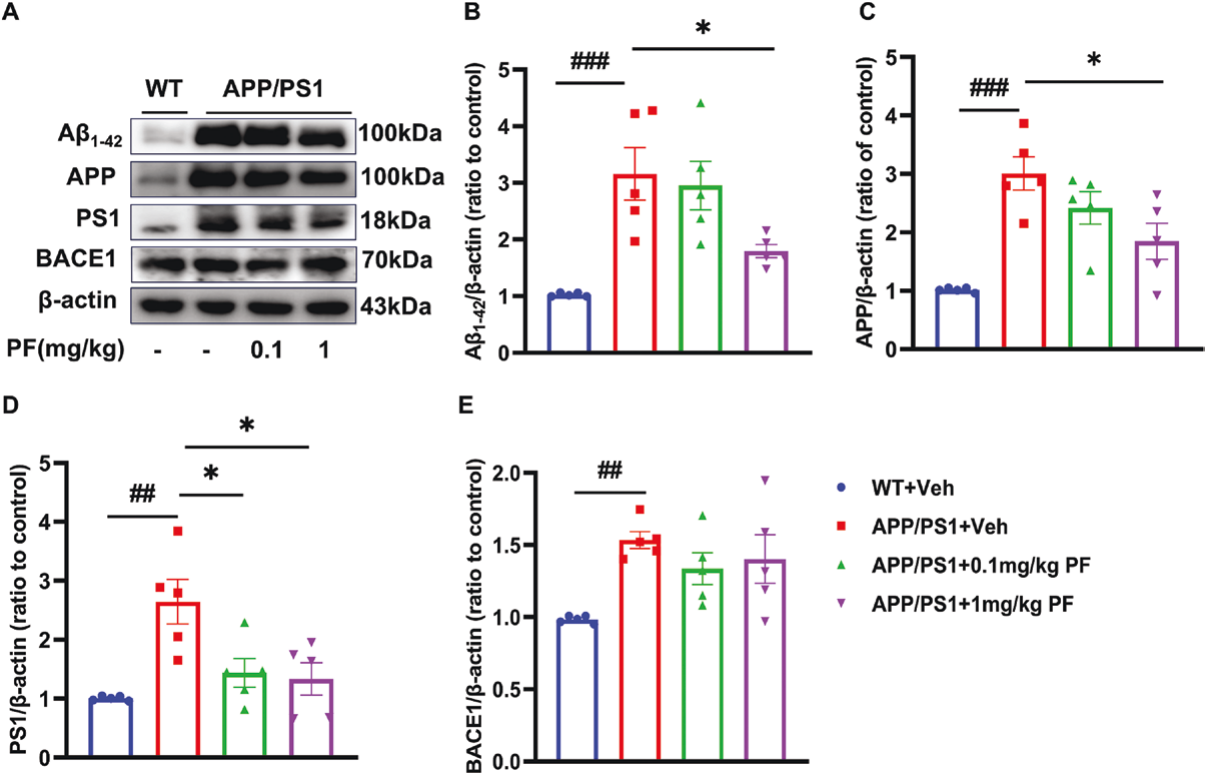
**PF reduced the production of Aβ**_**1-42**_ **in the hippocampus of APP/PS1 mice.** (A) Representative images by Western blotting showing the expression of Aβ_1-42_, APP, PS1, and BACE1 by PF pretreatment in APP/PS1 mice. (B-E) Quantitative analysis of Aβ_1-42_, APP, PS1, and BACE1, normalized by β-actin to WT with vehicle. The values shown are means ± SEM, normalized by β-actin to WT with vehicle. ^*##*^*P <* .01, ^###^*P <* .001vs WT plus vehicle. *P < .05 vs APP/PS1 plus vehicle; *n* = 5. APP, amyloid precursor protein; PF, PF-04957325; PS1, presenilin-1; WT, wild type.

As is well known, Aβ_1-42_ is the major component of senile plaques, which is one of the characteristic pathological features of AD. To further research the effect of inhibiting PDE8 on Aβ generation, the related protein expression of APP, PS1, BACE1 (Beta-Secretase 1), and Aβ_1-42_ was deleted by Western blotting. It was found that the expression of APP Aβ_1-42_, APP, PS1, and BACE1 in the hippocampus of APP/PS1 mice was significantly higher than that in WT mice (Figure 8A-E, *P <* .01). Furthermore, Aβ_1-42_, APP, and PS1 elevations in the hippocampus of APP/PS1 mice were suppressed by PF at 1 mg/kg (Figure 8A-D, *P <* .05), while the rise of PS1 expression also was suppressed by PF at 0.1 mg/kg (Figure 8A and D, *P <* .05).

## DISCUSSION

Microglia-mediated neuroinflammation is a key factor that promotes AD progression; inhibition of microglial over-activation has been shown to be a potential therapeutic strategy.^31^ The data from the present study demonstrated that PF inhibited AβO-induced microglial polarization in BV2 cells and reversed cognitive dysfunction in AD mice by inhibiting PDE8 and subsequently regulating downstream cAMP-related molecules. These results collectively highlight the clinical potential of PDE8 inhibitors for the management of AD.

Beta-amyloid is a key molecule implicated in AD pathogenesis.^32^ The amyloid hypothesis posits that AD can be caused by the accumulation of Aβ in the brain; immunotherapy targeting Aβ by encompassing anti-Aβ monoclonal antibodies has achieved clinical benefit, further validating Aβ as a therapeutic target for AD^.33^ According to earlier research, aggregated Aβ peptides activate microglial cells, the primary phagocytes in the CNS, to initiate inflammatory responses, including the production of inflammatory factors, eventually culminating in neurotoxicity and brain injury.^34-36^ Amyloid-β oligomers are considered more neurotoxic than Aβ fiber aggregates, with low concentrations of Aβ_1-42_ oligomers directly inducing neuronal damage.^37,38^ Consequently, AβO was used to induce the microglial activation in this study. Microglia are typically activated into a proinflammatory state or anti-inflammatory state. Different states can be identified by the expression of CD68, CD206, and Arg-1, respectively.^39,40^ As expected, microglial polarization was noted in AβO-treated BV2 cells. Specifically, the upregulated CD68 expression and downregulated CD206 and Arg-1 expression in AβO-exposed BV2 cells indicated a switch from an anti-inflammatory state to a proinflammatory state, resulting in the release of proinflammatory cytokines, including IL-6, IL-1β, TNF-α, iNOS, and COX-2. PDE8, as a hydrolytic enzyme that degrades the second messenger cAMP, has been found to be involved in the regulation of inflammation.^41,42^ It is found that PDE8 inhibitors exert regulatory effects on inflammation by inhibiting effector T cell (Teff) adhesion and proliferation and regulating Teff interactions with endothelial cells, which is beneficial in diseases such as asthma and chronic obstructive pulmonary disease.^25,41,43^ Meanwhile, PDE8 is ubiquitously expressed in the brain,^43^ and PDE8 inhibitor treatment attenuates the accumulation of both encephalitogenic Th1 and Th17 T cells in the CNS and thus inhibits inflammatory lesion formation, leading to beneficial profiles in CNS diseases such as multiple sclerosis,^23^ which suggest that PDE8 is also involved in the regulation of neuroinflammation. Microglia as the phagocyte of the CNS play an important role in the process of neuroinflammation.^44^ Research has shown that inhibiting PDE4 can alleviate neuroinflammation in BV2 microglial cells induced by lipopolysaccharide through the activation of cAMP/PKA/CREB signaling pathway,^45^ which indicates that PDE can serve as a target for controlling the activation of microglia and improving neuroinflammation through the regulation of cAMP. Meanwhile, our previous research found that PDE8 is distributed in the microglia of the brain of mouse,^44^ which suggests whether inhibition of PDE8 can also regulate microglial polarization and alleviated neuroinflammatory damage? That has been confirmed in this study. We found that the treatment with the PDE8 inhibitor PF suppressed the activation of proinflammatory microglia, promoted the transformation of anti-inflammatory microglia, and decreased the levels of these proinflammatory cytokines in vitro experiments. These results suggest that inhibiting PDE8 attenuates neuroinflammatory, and its mechanism may be related to the inhibition of microglial polarization.

To determine whether the anti-inflammatory effect of the PDE8 inhibitor contributed to the reversal of cognitive dysfunction and subsequent benefits for AD, we used 2 mouse models of AD, that is, AD mice generated by microinfusion of AβO into the hippocampus and APP/PS1 mice for behavioral tests. The Y-maze test, frequently conducted to assess short-term memory in mice, as well as the NOR and MWM, which are cognitive behavioral assays dependent on hippocampal function, were employed to assess the spatial learning and memory abilities of mice. The results demonstrated that PF resulted in significant improvements in cognitive function in both AD models, implying that inhibiting PDE8, similar to inhibiting PDE4 or PDE7^,17,46-49^ exerts anti-AD effects. However, further research on PDE8 is still needed because the understanding of its safety is still limited due to the lack of PDE8 inhibitors,^20^ especially when compared to some subtype selective PDE4 inhibitors with low emetic potential, such as PDE4B inhibitor A33.^50^ But PDE8 inhibitors can be still considered as a new opportunity to be explored as valuable drug candidates for AD. Meanwhile, the PDE8 inhibitor PF modulated microglial reprogramming, inhibited microglia with a proinflammatory state polarization, and limited the release of inflammatory cytokines in the hippocampus of AβO-induced AD model and APP/PS1 mice. Thus, we speculate that PDE8 inhibition may delay AD progression by modulating microglia-mediated inflammation. However, the upregulation of anti-inflammatory microglia induced by PF was only observed in the hippocampus of AβO-induced AD model mice, warranting further exploration. In addition, the present study also observed that the expression level of PDE8 was increased after AβO stimulation in both in vivo and in vitro experiments, suggesting that PDE8 may participate in neuroinflammation-induced Aβ, further corroborating our hypothesis. Of note, the PDE8 family comprises 2 subtypes, namely PDE8A and PDE8B, which have high affinity and specificity for cAMP.^51^ Herein, Aβ upregulated the expression of both subtypes of PDE8, illustrating that both PDE8A and PDE8B may play a decisive role in mediating inflammation. Nonetheless, further investigation on their pathophysiological relevance is necessitated.

Research shows that cAMP is degraded to adenosine monophosphate (AMP), thereby facilitating the production of proinflammatory mediators.^52,53^ Therefore, some PDE inhibitors function via inhibiting cAMP degradation, which may lead to a reduction in inflammation. PDE4 inhibitors such as roflumilast, apremilast, and crisaborole have been approved for the treatment of inflammatory airway diseases, psoriatic arthritis, and atopic dermatitis, respectively.^53^ Furthermore, PDE4 inhibitors such as roflumilast and rolipram can modulate neuroinflammation and drive the endogenous regeneration of neurons and oligodendrocytes, thereby delaying the progression of AD by governing cAMP and its downstream pathways.^54,55^ The PDE4D inhibitor zatolmilast has demonstrated positive effects in Phase II clinical trials for AD^.56^ Nevertheless, no effective PDE4 inhibitors are available for clinical use in AD. PDE8, PDE7, and PDE4 are all cAMP-specific hydrolases, and inhibition of PDE8 can also lead to the preservation of cAMP.^57^ Besides, our study here showed that the PDE8 inhibitor PF increased cAMP levels and promoted the phosphorylation of PKA and CREB, the downstream molecules of cAMP, in both cell and animal experiments.

BDNF, a neurotrophin, is a key molecule involved in plastic changes related to learning and memory^.58^ Although the exact mechanisms underlying the effect of impaired BDNF signaling on AD remain to be elucidated, mounting evidence suggests that decreased BDNF levels may contribute to Aβ accumulation, tau phosphorylation, neuroinflammation and neuronal apoptosis, and progressive atrophy of neurons in AD-affected brains.^59-61^ Previous studies have concluded that Aβ predominantly decreases BDNF levels by lowering phosphorylated CREB protein,^62^ in line with the results of cell and animal experiments in our study. Importantly, the PDE8 inhibitor PF significantly restored BDNF levels, indicative of PDE8 inhibition and exerting regulatory effects on BDNF by promoting CREB phosphorylation. Thus, BDNF upregulation may also be one of the mechanisms by which inhibition of PDE8 attenuates inflammation and exerts anti-AD effects. Furthermore, as is well known, BDNF is an important regulator of synaptic transmission and long-term potentiation (LTP) in the hippocampus and in other brain regions, playing a role in the formation of certain forms of memory.^63^ Meanwhile, it is now widely accepted that synaptic loss is a common hallmark of AD, while the overproduction of Aβ leads to synaptic dysfunction.^64^ So, in current research, 2 crucial synaptic proteins for the exocytosis and endocytosis of various neurotransmitters, SYP and PSD-95, were deleted to evaluate the damage induced by Aβ and the neuroprotective effects of PF. Our present data showed that both presynaptic and postsynaptic proteins, SYP and PSD-95, were downregulated in AβO-treated mice and APP/PS1 mice, while PF reversed these downregulations, indicating its improved effect on synaptic dysfunction. Although the detailed mechanism still needs further research, the current results indicated that the improvement of synaptic function is also one of the mechanisms underlying the anti-AD effect of PDE8 inhibition.

Preserving cAMP levels by inhibiting PDE can not only ameliorate Aβ-induced neuronal damage but also regulate Aβ generation.^63^ In this study, the PDE8 inhibitor PF decreased Aβ levels and downregulated the expression of APP and PS1 in APP/PS1 mice, suggesting that inhibiting Aβ generation may also be another mechanism by which PDE8 inhibition exerts favorable effects in AD. Nevertheless, it is acknowledged that our study has limitations. First, the relevant indicators in the Aβ model mice were assessed solely at protein levels. Future research could incorporate gene-level analyses, and positive control drugs may be employed to compare and assess efficacy. Additionally, this investigation is confined to the classical cAMP pathway; other signaling pathways merit further exploration in future studies.

## CONCLUSIONS

**Figure 9.**
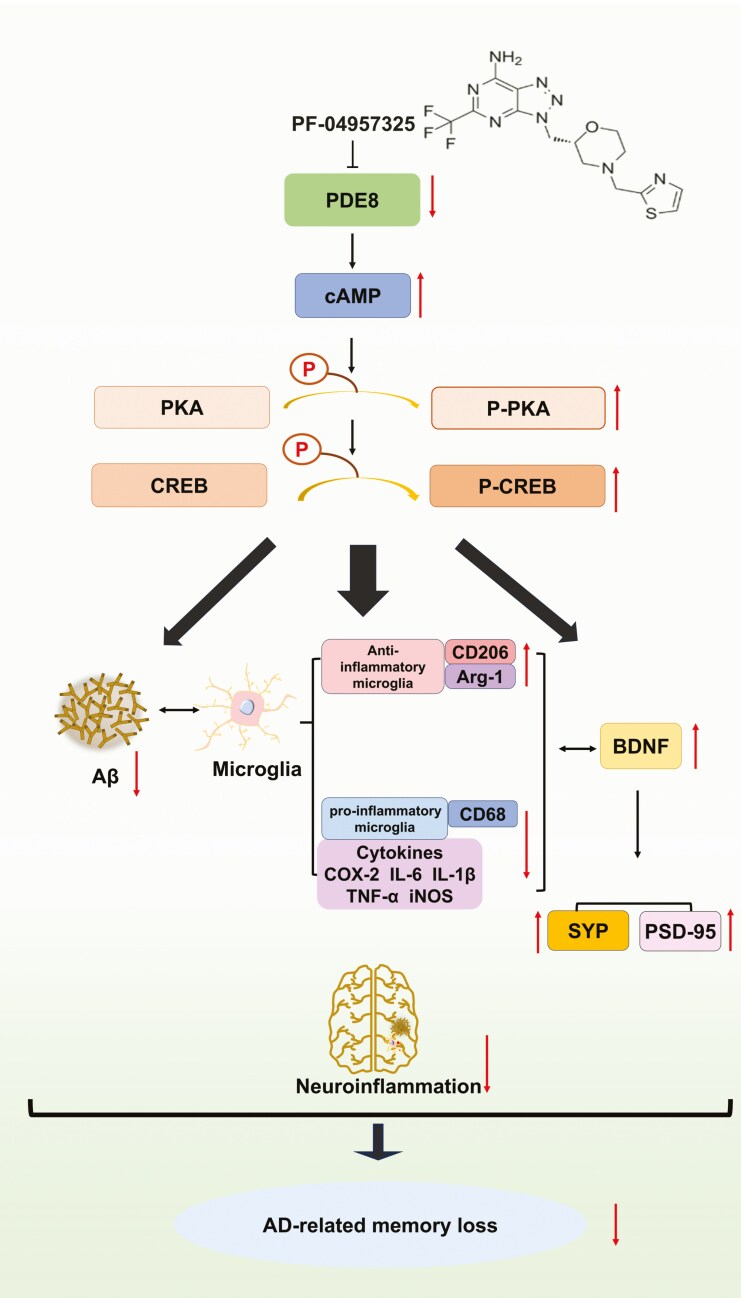
**PF ameliorates neuroinflammation induced by AβO.** PDE8 inhibitor alleviated AD through various mechanisms, including attenuating microglia-mediated neuroinflammation, upregulating the expression of BDNF, and inhibiting Aβ generation, which may be related to the PDE8/cAMP/CREB signaling pathway. AβO, amyloid-β oligomers; AD, Alzheimer’s disease; cAMP, cyclic adenosine monophosphate; BDNF, brain-derived neurotrophic factor; CREB, cAMP response element-binding protein; PDE8, phosphodiesterase-8; PF, PF-04957325.

In summary, our data uncovered that the PDE8 inhibitor PF alleviated AD-like changes in behavior and pathology through various mechanisms, including attenuating microglia-mediated neuroinflammation, upregulating the expression of BDNF, restoring synaptic dysfunction and inhibiting Aβ generation, which seem to be involved by the PDE8/cAMP/CREB signaling pathway. These results highlight the potential of PDE8 inhibitors as a novel class of therapeutic agents for AD (Figure 9).

## Data Availability

Datasets described in the manuscript may be available from the corresponding authors upon reasonable requests.

## Coonflicts of interest

None declared.

## Availability of data and materials

No datasets were generated or analyzed during the current study.

## Author details

Tian-yang Guo, Meng Zhang, and Yu-li Lv are co-first authors.
